# Intra-dialytic blood pressure variability is a greater predictor of cardiovascular events in hemodialysis patients

**DOI:** 10.1186/s12882-023-03162-w

**Published:** 2023-04-26

**Authors:** Qixing Liu, Wei Wang, Xianglan Wu, Jiaxuan Lv, Shiming Cai, Yuehong Li

**Affiliations:** 1 School of Medicine, Tsinghua University, Beijing, China; 2 Department of Nephrology, Beijing Tsinghua Changgung Hospital, School of Clinical Medicine, Tsinghua University, Beijing, China

**Keywords:** Hemodialysis, Blood pressure variability, Cardiovascular disease, All-cause mortality.

## Abstract

**Background:**

Short-term and long-term blood pressure variability (BPV) in hemodialysis (HD) population are risk factors of cardiovascular diseases (CVD) and all-cause mortality. There is no full consensus on the best BPV metric. We compared the prognostic role of intra-dialytic and visit-to-visit BPV metrics for CVD morbidity and all-cause mortality in HD patients.

**Methods:**

A retrospective cohort of 120 patients on HD was followed up for 44 months. Systolic blood pressure (SBP) and baseline characteristics were collected for 3 months. We calculated intra-dialytic and visit-to-visit BPV metrics, including standard deviation (SD), coefficient of variation (CV), variability independent of the mean (VIM), average real variability (ARV) and residual. The primary outcomes were CVD events and all-cause mortality.

**Results:**

In Cox regression analysis, both intra-dialytic and visit-to-visit BPV metrics were associated with increased CVD events (intra-dialytic CV: HR 1.70, 95% CI 1.28–2.27, p < 0.01; visit-to-visit CV: HR 1.55, 95% CI 1.12–2.16, p < 0.01), but not associated with increased all-cause mortality (intra-dialytic CV: HR 1.32, 95% CI 0.99–1.76, p = 0.06; visit-to-visit CV: HR 1.22, 95% CI 0.91–1.63, p = 0.18). Overall, intra-dialytic BPV showed greater prognostic ability than visit-to-visit BPV for both CVD event (AUC of intra-dialytic BPV and visit-to-visit BPV metrics respectively: SD 0.686, 0.606; CV 0.672, 0.425; VIM 0.677, 0.581; ARV 0.684, 0.618; residual 0.652, 0.586) and all-cause mortality (SD 0.671, 0.608; CV 0.662, 0.575; VIM 0.669, 0.581; ARV 0.529, 0.588; residual 0.651, 0.602).

**Conclusion:**

Compared to visit-to-visit BPV, intra-dialytic BPV is a greater predictor of CVD event in HD patients. No obvious priority was found among various BPV metrics.

**Supplementary Information:**

The online version contains supplementary material available at 10.1186/s12882-023-03162-w.

## Background

Hemodialysis (HD) population is under high risk of morbidity and mortality particularly due to cardiovascular disease (CVD) [1], which accounted for more than 50% deaths [2]. Blood pressure (BP) is a well-known contributor discovered to exhibit an inverse L- or U-shaped association with mortality [3]. However, growing evidences denote the shortcomings of the usual blood pressure hypothesis, and emphasize the importance of blood pressure variability (BPV) as an independent risk factor for cardiovascular morbidity and mortality [4]. And numerous studies have identified the association between BPV and cardiovascular mortality [5].

HD patients have higher BPV than the general population due to the routine removal of fluids and electrolytes during each dialysis session and volume-related progressive BP increase during the interdialytic interval [6]. According to different periods, BPV can be divided into very short-term BPV (beat-by-beat), short-term BPV (within 24 h), and long-term BPV (day-by-day or visit-to-visit) [7]. Short-term BPV and long-term BPV are most studied in HD patients. The two commonly-used approaches to calculate short-term BPV are based on intra-dialytic BP [8] and ambulatory BP monitoring [9, 10] representing BP fluctuation during dialysis and inter-dialysis respectively. The long-term BPV is usually generated by pre-dialytic BP [11]. Both short-term BPV [8–10] and long-term BPV [11–14] have been proved to associate with CVD morbidity, CVD mortality and all-cause mortality. Yet there is no full consensus on the best predictor.

Among all the studies mentioned above, several metrics representing variation were mainly implemented including standard deviation (SD) [9, 11], coefficient of variation (CV) [9, 11], variation independent of mean (VIM) [15, 16], average real variability (ARV) [10, 17] and residual [8, 14]. Although strong correlation was observed between these metrics, the clinical implication could be different. CV and VIM are transformation of SD which have reduced correlation with the mean, calculated by dividing SD by the mean or exponent of mean respectively. ARV emphasizes the difference between successive BP measurements. Residual takes the time influence on BP into account by fitting a mixed linear model. But the efficacy of different metrics has not been well-studied.

Although multiple studies have already proved the correlation between BPV and cardiovascular morbidity as well as all-cause mortality in HD patients, whether BPV is a modifiable risk factor remains undetermined and there are currently no guidelines for treatment recommend based on BPV [18]. Further research confirming these findings and investigating potential BPV mitigation strategies are required. In order to achieve this goal, determining a BPV metric easily acquired and tracked in the clinical setting is an essential step before BPV can be routinely monitored in the clinic [19]. But studies comparing short-term and long-term BPV metrics are still limited. Therefore, peri-dialytic BP routinely recorded in the HD center is used for this study, and the main purpose is to evaluate the prognostic ability of intra-dialytic and visit-to-visit BPV metrics for CVD events and all-cause mortality.

## Methods

### Study Design

This was a retrospective study. The inclusion criteria were as followed: (1) patients with end-stage renal disease (ESRD) from the Dialysis Center at Tsinghua Changgung Hospital between January 1st 2018 and Dec 31st 2018; (2) aged ≥ 18 years old; (3) on maintenance HD for more than 3 months in 2018. Patients were excluded under the following circumstances: (1) had no available BP measurement data more than 3 months in 2018; (2) died or transferred to another dialysis center before Dec 31st 2018. Finally, a total of 120 patients were included in the cohort.

### BPV metrics and covariates

Data was extracted from the electronic medical record from Tsinghua Changgung Hospital according to the organization’s standard clinical protocols. HD patients received regular HD thrice-weekly (on the 1st, 3rd, 5th or 2nd, 4th, 6th day of the week), and each dialysis session generally lasted for 4 h. All BP during each dialysis session in our Center was routinely and automatically measured by HD machine in a consensus manner (before, after, and during each dialysis session typically at 1 h intervals, total 5 SBP measurements per patient per dialysis session). The patient was in a resting state and lying position, and the BP on the non-fistula arm was measured. The peri-dialytic SBP during the 3 months exposure period for each patient (started from the date of the first dialysis session in 2018) was used for the calculation of BPV metrics (4 weeks per months, 3 dialysis sessions per week, total about 36 dialysis sessions). Intra-dialytic BPV (refer to variation of BP within dialysis sessions) and visit-to-visit BPV (refer to variation of pre-dialytic BP across dialysis sessions) were selected as indicators of short-term BPV and long-term BPV, respectively. All 5 SBP measures of dialysis sessions were used for intra-dialytic BPV, while only the pre-dialytic SBP of dialysis sessions were used for visit-to-visit BPV.

As for intra-dialytic BPV, five different metrics were analyzed using peri-dialysis SBP: (1) standard deviation (SD); (2) coefficient of variation (CV), calculated as SD divided by the mean and multiplied by 100%; (3) variability independent of mean (VIM), calculated as: $VIM=k\times SD/{\stackrel{-}{x}}^{m}$, where k and m are constants acquired by fitting a power model $SD=constant\times {\stackrel{-}{x}}^{m}$ and $k={\stackrel{=}{x}}^{m}$ [15]; (4) average real variability (ARV), which is the mean of absolute differences between successive SBP readings during each dialysis session [20]; (5) average residual, estimated by fitting a 2-slope mixed linear model over time [8]. As for visit-to-visit BPV, pre-dialysis SBP of each dialysis session was used to calculate SD, CV, VIM, ARV and residual. The visit-to-visit residual was estimated by fitting a mixed-effects linear model over time [11, 14]. Detailed calculation was shown in Supplementary Material.

Data for covariates were collected from electronic clinical records, including demographic characteristics (age, gender), personal history (smoking history, drinking history), comorbidities (diabetes, hypertension, hyperlipidemia, CVD history, malignant tumor), dialysis-related variables (dialysis vintage, Kt/V, ultrafiltration volume, dry weight) and laboratory parameters (hemoglobin, albumin, creatinine, blood urea nitrogen, potassium, phosphorus, calcium, parathyroid hormone, total cholesterol, low density lipoprotein). Patients with any of the following conditions (coronary heart disease, heart failure, previous stroke, peripheral vascular disease) were considered to have CVD history. Laboratory parameters were measured monthly. The average values during the exposure period served as the baseline data.

### Outcomes

Our primary outcomes were CVD events and all-cause mortality. The follow-up period was from Jan 1st 2019 to Aug 31st 2022. CVD events include heart failure, myocardial infarction, ventricular arrhythmias, cerebral infarction, cerebral hemorrhage, and peripheral artery disease (PAD) required surgical intervention. CVD events were acquired from hospitalization records and reviewed by physicians to ascertain. Deaths were identified from death certificates of hospital records. Participants were censored at the following circumstances: (1) received a kidney transplant; (2) transferred to another dialysis facility; (3) come to the end of the follow-up period.

### Statistical methods

Continuous variables were described by mean and SD, while categorical variables were described by frequency and proportion. Spearman’s correlation test was performed in order to evaluate the association between BPV metrics and covariates.

The BP data from each patient were analyzed separately as intradialytic data and visit-to-visit BP data. Patients were divided into two groups based on their intra-dialytic or visit-to-visit CV, and the mean CV were used as cutoff values. The high CV group was defined as greater than or equal to the mean CV. The low CV group was defined as less than the mean CV. Variables were compared between the high and low CV groups using Wilcoxon rank sum test. Kaplan-Meier estimator was used to calculate the cumulative incidence of cardiovascular events and all-cause death. Log-rank test was subject to compare survival between the two groups.

To further assess the potential association of BPV with CVD morbidity and all-cause mortality, unadjusted (containing only one of the BPV metrics) and adjusted Cox proportional hazards models were conducted. Covariates plausibly associated with CVD morbidity or all-cause mortality (defined as P < 0.1 in univariate cox hazard analysis) were selected for the adjustment: age, gender, smoking history, drinking history, diabetes, hyperlipidemia, CVD history, tumor, Kt/V, albumin, hemoglobin and creatinine. Phosphorus and ultrafiltration volume were also adjusted. The process deciding confounders is shown in Table S3 (Supplementary Material). Receiver operating characteristic (ROC) curves were applied to compare the prognostic ability of different BPV metrics. Bootstrap test or DeLong’s test was used for comparing ROC curves.

All analyses were performed using R [21]. The R package survival was used to perform Cox regression analysis [22]. The R package pROC was used to conduct ROC curve plotting and analysis [23]. Statistical significance was defined as two-tailed p-values < 0.05.

## Results

### Cohort characteristics

There were 218 patients underwent maintenance HD in Dialysis Center of Tsinghua Changgung Hospital since January 1st 2018. After excluding by less than 3 months of HD (n = 81), death (n = 9) and transfer to another dialysis center (n = 8) in 2018, total 120 adult patients on regular HD were enrolled in the cohort. During the 3 months exposure period, 4386 HD treatments and 21,408 SBP measurements were recorded. Table 1 presents demographic, personal history, dialysis-related variables and laboratory parameters of the cohort. Patients aged 61.43 ± 14.26 years and composed of 61.7% male were treated with HD for 1327 ± 402 days. The SBP was 143.04 ± 16.92 mmHg, and the DBP was 72.61 ± 9.44 mmHg. The prevalence of comorbidities included 50.8% diabetes, 50.8% hyperlipidemia, 85.8% hypertension, 49.2% cardiovascular disease and 15.8% malignant tumor. The follow-up time were 898.1 ± 419.8 days for CVD events, and 1008.3 ± 394 days for all-cause mortality.

**Table 1:** Baseline characteristics of HD patients

Parameter	Value
Sample size	120
Male, n (%)	74 (61.7)
Age (years)	61.43 ± 14.26
SBP (mmHg)	143.04 ± 16.92
DBP (mmHg)	72.61 ± 9.44
Smoking habit, n (%)	32 (26.7)
Drinking habit, n (%)	9 (7.5)
Comorbidities, n (%) DM	61 (50.8)
Hyperlipidemia	61 (50.8)
Hypertension	103 (85.8)
Cardiovascular disease	59 (49.2)
Malignant tumor	19 (15.8)
Dialysis parameters	
UF (L)	2.05 ± 0.68
Dry weight (kg)	64.92 ± 11.49
Kt/V	1.41 ± 0.26
Dialysis vintage (days)	1327.63 ± 402.83
Laboratory parameters	
Alb (g/L)	39.53 ± 3.09
Hb (g/L)	113.12 ± 13.74
Cr (umol/L)	857.85 ± 257.58
BUN (mmol/L)	26.03 ± 5.37
K (mmol/L)	5.08 ± 0.72
Ca (mmol/L)	2.17 ± 0.16
P (mmol/L)	1.81 ± 0.46
PTH (pg/mL)	227.40 ± 152.72
TC (mmol/L)	3.88 ± 0.90
LDL (mmol/L)	1.95 ± 0.70

Continuous variables were presented as mean ± SD; categorical variables were presented as n (%). SBP, systolic blood pressure; DBP, diastolic blood pressure; DM, diabetes mellitus; UF, ultrafiltration; Alb, albumin; Hb, hemoglobin; Cr, creatinine; BUN, blood urea nitrogen; K, potassium; Ca, calcium; P, phosphorus; PTH, parathyroid hormone; TC, total cholesterol; LDL, low density lipoprotein

**Fig. 1:**
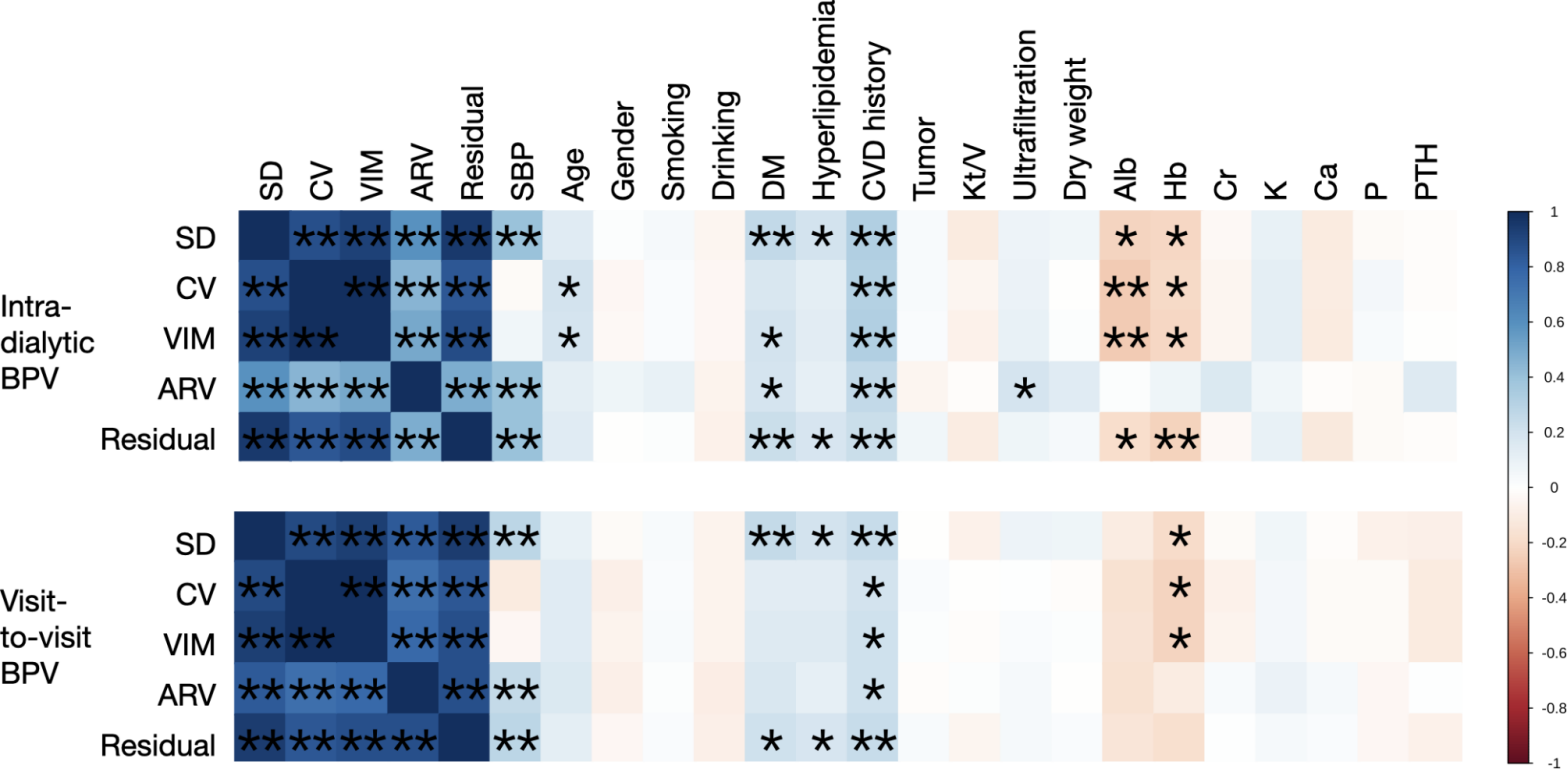
Association between intra-dialytic/visit-to-visit BPV and covariates Spearman’s correlation test was performed to evaluate the correlation between BPV metrics and covariates. Color represents coefficient. ‘**’ indicates p<0.01, ‘*’ indicates p<0.05

### Association between BPV and covariates

Table 2 demonstrates the intra-dialytic and visit-to-visit BPV metrics. Association between BPV and covariates are plotted in Fig. 1, and coefficient is described in Table S1 (Supplementary Material). The BPV metrics were highly correlated with each other. SD, ARV and residual show correlation with SBP, whereas CV and VIM were independent of SBP. CVD history was correlated with all BPV metrics. Diabetes and hyperlipidemia were correlated with part of the intra-dialytic and visit-to-visit BPV metrics. Only intra-dialytic ARV manifested association with ultrafiltration volume. Correlation with BPV was also identified in albumin and hemoglobin levels.

**Table 2:** BPV metrics of HD patients

BPV metrics	Mean ± SD
Intra-dialytic BPV	
SD (mmHg)	15.42 ± 4.07
CV (%)	11.11 ± 2.77
VIM (unit)	15.39 ± 3.80
ARV (mmHg)	10.66 ± 2.96
Residual (mmHg)	11.28 ± 3.08
Visit-to-visit BPV	
SD (mmHg)	14.47 ± 4.38
CV (%)	10.15 ± 2.89
VIM (unit)	14.45 ± 4.12
ARV (mmHg)	14.50 ± 4.68
Residual (mmHg)	10.80 ± 3.46

### Comparison of covariates and outcomes between high and low CV groups

**Fig. 2:**
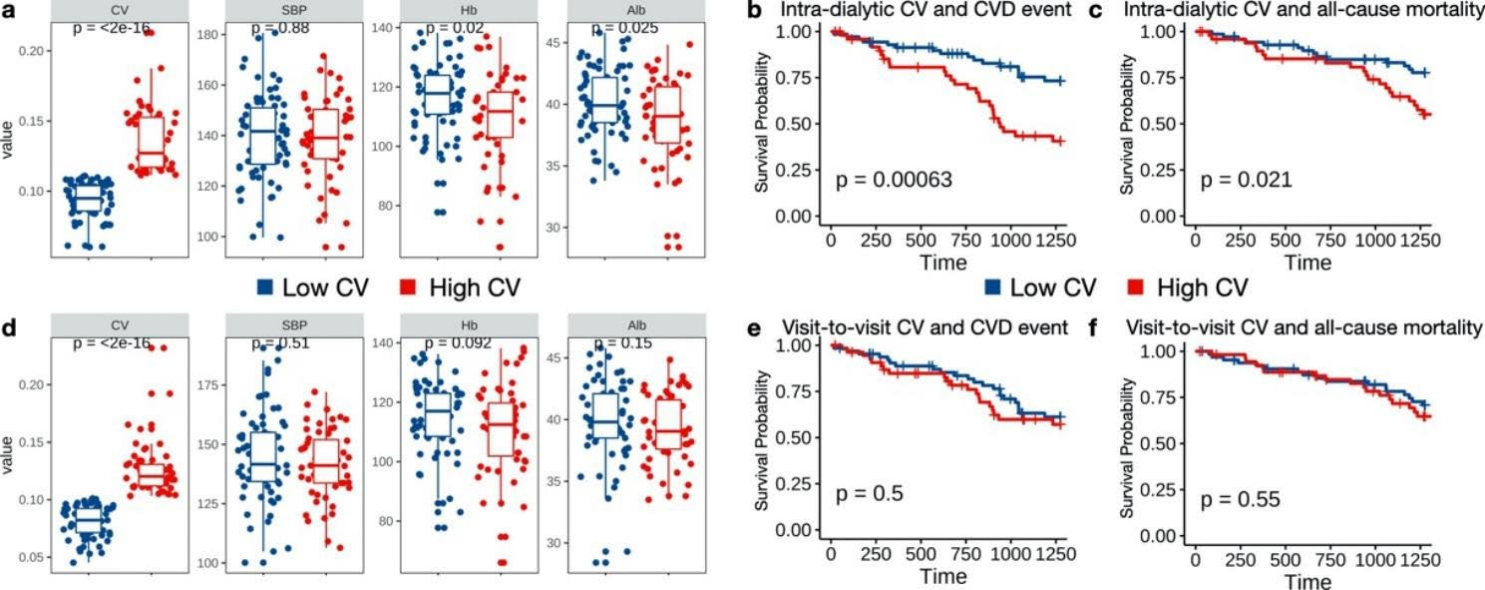
Comparison of covariates and outcomes between high and low CV groups HD patients were divided into two groups based on intra-dialytic CV (**a**–**c**) and visit-to-visit CV (**d**–**f**). (**a**, **d**) Boxplot of CV, SBP, Hb and Alb. P value was calculated by Wilcoxon rank sum test. (**b**, **e**) Kaplan-Meier survival plots of CVD morbidity. (**c**, **f**) Kaplan-Meier survival plots of all-cause mortality. P value was calculated by log-rank test

Patients were divided into two groups based on the cutoff of the mean intra-dialytic or visit-to-visit CV separately (mean intra-dialytic CV = 11.11%; mean visit-to-visit CV = 10.15%; high CV group: CV ≥ mean CV; low CV group: CV < mean CV). Characteristics of high and low CV groups were shown in Table S2 (Supplementary Material). SBP was not different between the two groups. Hemoglobin and albumin were significantly decreased in the high intra-dialytic CV group (Fig. 2a) but not in the high visit-to-visit CV group (Fig. 2d) compared with corresponding low CV group. High intra-dialytic CV group had increased CVD morbidity and all-cause mortality compared with low intra-dialytic CV group (Fig. 2b, c), but visit-to-visit CV groups had no such difference (Fig. 2e, f).

### Association between BPV and outcomes

During follow-up, 42 patients encountered CVD event, and overall CVD morbidity was 35%, including 28 cardiac diseases (66.7%), 7 strokes (16.7%), and 7 peripheral vascular disease (16.7%). 35 deaths occurred in 331.5 patient-years of follow-up. The mortality rate was 105.6 deaths/1000 patient-years. 14 deaths (40%) were attributed to cardiovascular causes. Infection and tumor were the other two main causes accounting for 6 (17%) and 7 deaths (20%) respectively.

Crude and adjusted hazard ratios (HRs) for CVD event and all-cause mortality were shown in Table 3. Complete results of adjusted model for intra-dialytic and visit-to-visit CV were shown in Table S4 (Supplementary Material). Higher intra-dialytic and visit-to-visit BPV metrics were associated with increased CVD morbidity. Among intra-dialytic BPV metrics, CV had the highest crude HR of 1.703 (95%CI 1.281–2.265, P = 0.0003) and adjusted HR of 1.801 (95%CI 1.224–2.649, P = 0.0028) for the occurrence of CVD events. As for visit-to-visit BPV metrics, ARV had the highest crude HR of 1.638 (95%CI 1.182–2.270, P = 0.0031) and adjusted HR of 1.674 (95%CI 1.139–2.460, P = 0.0088) for the CVD morbidity. As regards of association with all-cause mortality, intra-dialytic SD exhibited a significant crude HR of 1.346 (95%CI 1.011–1.791, P = 0.0415) and adjusted HR of 1.455 (95%CI 0.990–2.137, P = 0.0562). After adjustment, none of the BPV metrics showed significant association with all-cause mortality.

**Table 3:** Association between intra-dialytic/visit-to-visit BPV and CVD morbidity and all-cause mortality

	Crude hazard ratio		Adjusted hazard ratio	
	HR (95%CI)	p-value	HR (95%CI)	p-value
**Intra-dialytic BPV and CVD event**				
SD	1.617 (1.225–2.133)	0.0007	1.726 (1.205–2.473)	0.0029
CV	1.703 (1.281–2.265)	0.0003	1.801 (1.224–2.649)	0.0028
VIM	1.696 (1.278–2.250)	0.0003	1.808 (1.234–2.649)	0.0024
ARV	1.465 (1.146–1.874)	0.0023	1.557 (1.163–2.086)	0.0030
Residual	1.476 (1.116–1.952)	0.0063	1.551 (1.103–2.181)	0.0116
**Intra-dialytic BPV and All-cause mortality**				
SD	1.346 (1.011–1.791)	0.0415	1.455 (0.990–2.137)	0.0562
CV	1.317 (0.985–1.761)	0.0632	1.282 (0.844–1.950)	0.2445
VIM	1.325 (0.993–1.767)	0.0557	1.320 (0.874–1.995)	0.1872
ARV	1.103 (0.790–1.541)	0.5640	1.256 (0.863–1.828)	0.2340
Residual	1.258 (0.942–1.680)	0.1200	1.312 (0.906–1.899)	0.1507
**Visit-to-visit BPV and CVD event**				
SD	1.507 (1.111–2.044)	0.0084	1.596 (1.083–2.353)	0.0181
CV	1.554 (1.120–2.156)	0.0083	1.476 (0.990–2.200)	0.0559
VIM	1.552 (1.124–2.143)	0.0076	1.511 (1.013–2.254)	0.0429
ARV	1.638 (1.182–2.270)	0.0031	1.674 (1.139–2.460)	0.0088
Residual	1.396 (1.063–1.834)	0.0165	1.431 (1.002–2.043)	0.0487
**Visit-to-visit BPV and All-cause mortality**				
SD	1.210 (0.914–1.602)	0.1840	1.224 (0.835–1.795)	0.3008
CV	1.220 (0.911–1.633)	0.1830	1.134 (0.750–1.716)	0.5512
VIM	1.216 (0.912–1.623)	0.1830	1.151 (0.765–1.730)	0.5007
ARV	1.156 (0.859–1.556)	0.3390	1.267 (0.860–1.866)	0.2322
Residual	1.193 (0.900-1.583)	0.2200	1.182 (0.808–1.729)	0.3901

### Comparison of ROC curves between BPV metrics

**Fig. 3:**
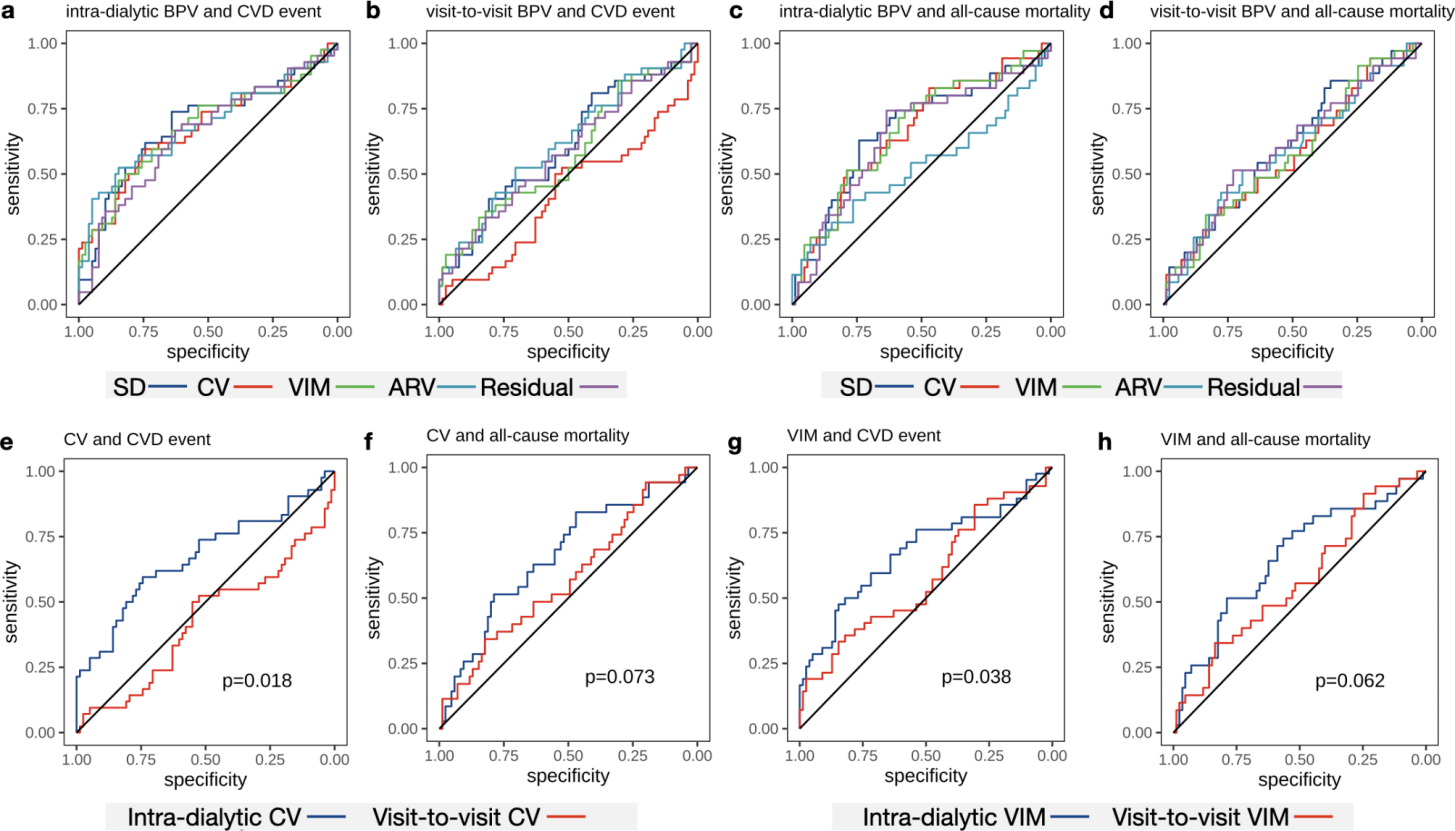
Comparison of ROC curves between BPV metrics ROC curves for CVD event predicted by intra-dialytic BPV (**a**) and visit-to-visit BPV (**b**). ROC curves for all-cause mortality predicted by intra-dialytic BPV (**c**) and visit-to-visit BPV (**d**). Comparison of ROC curves between intra-dialytic CV and visit-to-visit CV for CVD event (**e**), and for all-cause mortality (**f**). Comparison of ROC curves between intra-dialytic VIM and visit-to-visit VIM for CVD event (**g**), and for all-cause mortality (**h**). P value was calculated by Bootstrap test or DeLong’s test

Area under curve (AUC) of unadjusted Cox regression model was presented in Table 4. Receiver operating characteristic (ROC) curves of BPV metrics were plotted (Fig. 3a-d). Among intra-dialytic BPV metrics, SD showed better performance than residual in predicting CVD events (p = 0.029), while no difference was discovered in the rest of metrics (SD vs. CV, p = 0.62; SD vs. VIM, p = 0.70; SD vs. ARV, p = 0.97; CV vs. VIM, p = 0.47; CV vs. ARV, p = 0.85; CV vs. residual, p = 0.52; VIM vs. ARV, p = 0.91; VIM vs. residual, p = 0.35; ARV vs. residual, p = 0.58). Intra-dialytic SD and VIM outperformed ARV in predicting all-cause mortality (p = 0.017, 0.036). No significant difference of ROC curves between visit-to-visit BPV metrics was discovered. All intra-dialytic BPV metrics had higher AUC than corresponding visit-to-visit BPV metrics in the prediction of CVD event and all-cause mortality except ARV, which had an opposite tendency in prediction of all-cause mortality. Intra-dialytic CV and intra-dialytic VIM showed significantly increased prognostic ability in prediction of CVD event (Fig. 3e, g) and a trend of improvement in prediction of all-cause mortality (Fig. 3f, h), comparing with visit-to-visit CV and VIM respectively.

**Table 4:** AUC of BPV metrics

	Intra-dialytic BPV	Visit-to-visit BPV
	AUC (95%CI)	AUC (95%CI)
**CVD event**		
SD	0.686 (0.578–0.793)	0.606 (0.497–0.714)
CV	0.672 (0.563–0.780)	0.425 (0.313–0.538)
VIM	0.677 (0.568–0.785)	0.581 (0.470–0.692)
ARV	0.684 (0.574–0.793)	0.618 (0.510–0.725)
Residual	0.651 (0.543–0.759)	0.586 (0.476–0.696)
**All-cause mortality**		
SD	0.671 (0.558–0.784)	0.608 (0.497–0.719)
CV	0.662 (0.553–0.771)	0.575 (0.461–0.690)
VIM	0.669 (0.559–0.778)	0.581 (0.468–0.695)
ARV	0.529 (0.402–0.657)	0.588 (0.473–0.704)
Residual	0.650 (0.537–0.764)	0.602 (0.487–0.717)

## Discussion

In this study, we recruited total 120 patients on maintenance HD as a retrospective cohort, and calculated the intra-dialytic as well as visit-to-visit BPV metrics using peri-dialytic SBP during the 3 months exposure period. We found both intra-dialytic and visit-to-visit BPV were associated with CVD morbidity but not with all-cause mortality. Overall, intra-dialytic BPV outperformed visit-to-visit BPV in prediction of CVD event. Although without reaching significance, intra-dialytic BPV also showed better performance in prediction of all-cause mortality than visit-to-visit BPV.

There are already multiple studies that proved both short-term BPV [8–10] and long-term BPV [11–14] are associated with CVD morbidity, CVD mortality and all-cause mortality. But none of them compared the prognostic ability of adverse outcomes between short-term BPV and long-term BPV. Hence, we covered all the commonly used metrics for intra-dialytic and visit-to-visit BPV, including SD, CV, VIM, ARV and residual, in order to obtain a comprehensive comparison. In the prediction of CVD event, intra-dialytic BPV metrics were better predictors with higher AUC than visit-to-visit BPV. Across intra-dialytic BPV metrics, no significant difference was found (except residual was inferior to SD, p = 0.029). Novel metrics such as VIM, ARV and residual didn’t show a significantly better performance than conventional metrics. With additional consideration of independency from SBP and simplicity of calculation, intra-dialytic CV could be a relatively favorable target. Moreover, intra-dialytic ARV was the only metric showing correlation with ultrafiltration volume in our study, implying its unique characteristic and potential application value. Similar association between intra-dialytic ARV and fluid removal was also discovered in other study [24]. Intra-dialytic BPV also had better performance in prediction of all-cause mortality than visit-to-visit BPV. But the limitation was that BPV does not significantly associate with all-cause mortality in our study.

Short-term BPV reflects the various physiologic mechanisms, such as autonomic modulation, arterial stiffness, humoral response, blood viscosity, behavior, emotional and environmental factors, whereas long-term BPV additionally involves compliance with prescribed therapeutic regimen and seasonal climate changes [7]. In HD patients, the dialysis-related and interdialytic factors should also be considered, including UF-driven fluid shifts, serum osmolality changes, dialytic removal of antihypertensives and vasoactive substances [25], and interdialytic fluid accumulation [9]. Although it is pointless to identify precise contribution of distinct mechanisms in clinical setting, it helps us to better understand the BPV.

Greater prognostic ability of intra-dialytic BPV may suggest a major role of dialysis-related factors causing adverse outcomes. Dialysis could be considered as a shock due to the rapid removal of fluids and electrolytes, which may better reflect the body’s ability to regulate BP under stress, like a cardiac stress test for heart evaluation. The limitation of intra-dialytic BPV is that it can’t reflect influence out of dialysis unit such as fluid accumulation, circadian fluctuation and behavior changes. Moreover, peri-dialytic SBP is not the only choice to evaluate BPV. Ambulatory SBP and home SBP measurement have shown superior risk prediction for adverse outcomes [26–28]. And BPV derived from ambulatory and home SBP measurements manifested association with CVD morbidity and all-cause mortality as well [28]. The BPV based on 44-h ambulatory monitoring during interdialytic interval has been proved to predict outcomes [9]. Here, we didn’t include ambulatory and home SBP, because these two measurements have not been routinely monitored in clinic yet. Further investigation comparing between BPV derived from various measurements is required. Perhaps metrics, obtained by combination of intra-dialytic and inter-dialytic BPV, can be explored in the future.

Our study comprehensively compared the prognostic ability of intra-dialytic and visit-to-visit BPV metrics in predicting CVD events and all-cause mortality, which provides evidence for selecting the most efficient BPV metric. Determining a widely accepted and efficient BPV metric is the first step to build a BPV-based guideline for HD population. Non-pharmacologic treatments, such as restricting sodium intake and dialysis prescription, and antihypertensive medications are main approaches to manage BP and volume in HD patients [18]. Nevertheless, studies investigating the influence of these interventions on BPV are scarce. Our work can also provide reference for reliable BPV evaluation in the further research. However, there were also some limitations in our study. First of all, this was a single center retrospective study and the cohort size was limited, which could cause bias. Second, there were possible confounding factors that we didn’t include, such as anti-hypertensive medication, and the malnutrition, inflammation, and atherosclerosis (MIA) syndrome [29]. The high intra-dialytic CV group showed lower Alb and Hb, indicating MIA syndrome might contribute to the higher adverse outcomes. And we could not exclude the role of medication as well. Finally, our conclusion for BPV was based on an exposure period for 3 months. Different durations might also affect the results.

## Conclusions

In conclusion, this study showed that both intra-dialytic and visit-to-visit BPV were associated with risk of CVD events in HD patients. Overall, intra-dialytic BPV performed better prognostic ability than visit-to-visit BPV in prediction of CVD morbidity, suggesting intra-dialytic BPV to be a better predictor in HD population.

## Electronic supplementary material

Below is the link to the electronic supplementary material.

Supplementary Material 1

## Data Availability

The datasets generated and/or analyzed during the current study are available from the corresponding author on reasonable request.

## List of abbreviations

HD: Hemodialysis
CVD: Cardiovascular disease
BP: Blood pressure
SBP: Systolic blood pressure
DBP: Diastolic blood pressure
BPV: Blood pressure Variability
SD: Standard deviation
CV: coefficient of variation
VIM: variation independent of mean
ARV: Average real variability
ESRD: End-stage renal disease
PAD: Peripheral artery disease
ROC: Receiver operating characteristic
DM: Diabetes mellitus
UF: Ultrafiltration
Alb: Albumin
Hb: Hemoglobin
Cr: Creatinine
BUN: Blood urea nitrogen
K: Potassium
Ca: Calcium
P: Phosphorus
PTH: Parathyroid hormone
TC: Total cholesterol
LDL: Low density lipoprotein
HRs: hazard ratios
AUC: Area under curve
