# Supplementary material for: Intra-dialytic blood pressure variability is a greater predictor of cardiovascular events in hemodialysis patients
Source: BMC Nephrol. 2023 Apr 26;24:113. doi: 10.1186/s12882-023-03162-w (PMC10134565; doi:10.1186/s12882-023-03162-w)
Supplement: Supplementary file 1 — Supplementary Material 1 [file 12882_2023_3162_MOESM1_ESM.docx]

# Intra-dialytic Blood Pressure Variability Is a Greater Predictor of Cardiovascular Events in Hemodialysis Patients

Qixing Liu^1^, Wei Wang^2^, Xianglan Wu^2^, Jiaxuan Lv^2^, Shiming Cai^2^, Yuehong Li^2^.

Corresponding author: Yuehong Li, Email: lyha01051@btch.edu.cn.

Affiliation:

1 School of Medicine, Tsinghua University, Beijing, China.

2 Department of Nephrology, Beijing Tsinghua Changgung Hospital, School of Clinical Medicine, Tsinghua University, Beijing, China.

# Supplementary Material

### Calculation of BPV metrics

Intra-dialytic SD is calculated as follows, where SBP represents peri-dialytic SBP among the 3 months exposure period, n represents the number of peri-dialytic SBP readings. The schematic plot is shown in Supplementary Fig. 1a.

$$SD= \sqrt{\frac{\sum_{i=1}^{n} \left( {SBP}_{i}-\bar{SBP} \right)^{2}}{n-1}}$$

Intra-dialytic CV is calculated by dividing intra-dialytic SD by the mean peri-dialytic SBP and multiplying by 100%.

$$CV=\frac{SD}{\bar{SBP}}\times100\%$$

Intra-dialytic VIM is calculated by the equation shown below. m is a constant acquired by fitting a power model as follows. k is also a constant calculated from m. SD represents intra-dialytic SD, $\bar{SBP}$ represents the mean peri-dialytic SBP for each patient, and $SBP$ is the mean peri-dialytic SBP of all patients. As for intra-dialytic VIM in this study, k=61.04278, m=0.8337003.

$$VIM=k\times{SD}/{\bar{SBP}^{m}}$$

$$SD=constant\times\bar{SBP}^{m}$$

$$k={SBP}^{m}$$

Intra-dialytic ARV is the average ARV of all dialysis sessions during the exposure period. The calculation of ARV is shown as follows. SBP_k_ means the k^th^ reading of SBP, and n means the number of readings for each session. The schematic plot is shown in Supplementary Fig. 1b.

$$ARV= \frac{\sum_{k=1}^{n-1} |{SBP}_{k+1}-{SBP}_{k}|}{n-1}$$

Intra-dialytic residual is the average residual of peri-dialytic SBP readings from the predicted values estimated by fitting a 2-slope mixed-effects linear model with main effect terms for the time during a dialysis session. A typical dialysis session lasts for 4h. Hence, we decided to set the knot at 1h (at 0.25 proportion of HD elapsed). The schematic plot is shown in Supplementary Fig. 1c.

Visit-to-visit SD is calculated using pre-dialytic SBP among the 3 months exposure period. The calculation is the same as intra-dialytic SD. The schematic plot is shown in Supplementary Fig. 1d.

Visit-to-visit CV is calculated by visit-to-visit SD divided by the mean pre-dialytic SBP.

Visit-to-visit VIM is calculated using the same equation and model as intra-dialytic VIM. SD represents visit-to-visit SD, $\bar{SBP}$ represents the mean pre-dialytic SBP for each patient, and $SBP$ is the mean pre-dialytic SBP of all patients. As for visit-to-visit VIM in this study, k=56.61194, m=0.8138768.

Visit-to-visit ARV is the ARV of pre-dialytic SBP during the exposure period. The equation is the same as that for intra-dialytic ARV. SBP_k_ means the pre-dialytic SBP of k^th^ dialysis session, and n means the number of dialysis sessions. The schematic plot is shown in Supplementary Fig. 1e.

Visit-to-visit residual is the average residual of pre-dialytic SBP readings from the predicted values estimated by a mixed-effects linear model over time. The schematic plot is shown in Supplementary Fig. 1f.


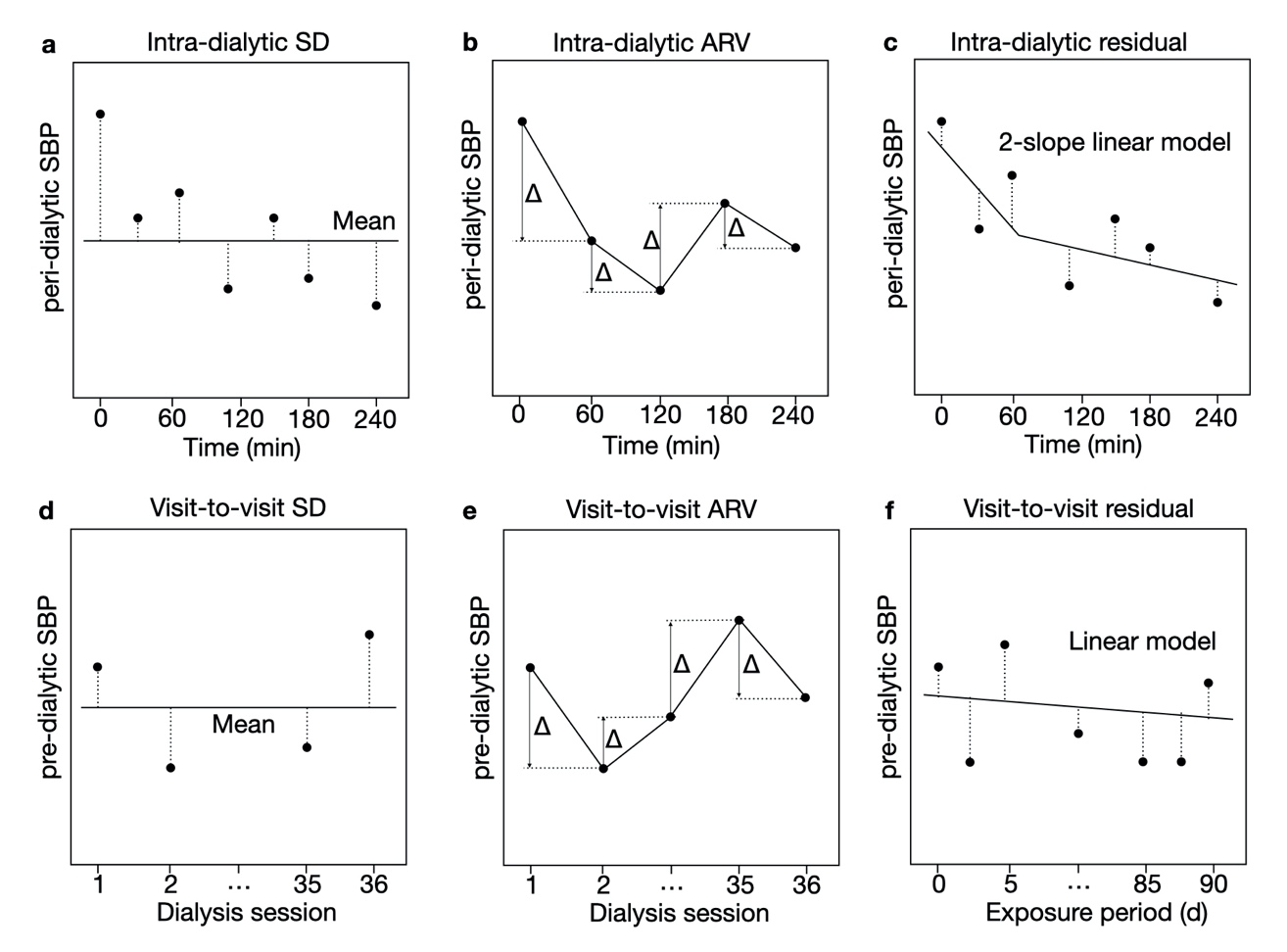


**Supplementary Fig. 1** Schematic diagram of BPV metrics

(a) intra-dialytic SD, the dashed line represents the deviation of each reading from the mean peri-dialytic SBP. (b) intra-dialytic ARV, Δ represents the deviation between consecutive peri-dialytic SBP readings. (c) intra-dialytic residual, the solid line represents a 2-slope mixed-effects linear model with a knot at 60min. The dashed line demonstrates the residual of peri-dialytic SBP readings from the predicted values using a 2-slope. (d) visit-to-visit SD, the dashed line represents the deviation of pre-dialytic SBP from the mean pre-dialytic SBP. (e) visit-to-visit ARV, Δ represents the deviation between consecutive pre-dialytic SBP readings. (f) visit-to-visit residual, the solid line represents a mixed-effects linear model over time. The dashed line demonstrates the residual of pre-dialytic SBP readings.

**Table S1** Correlation coefficient between intra-dialytic/visit-to-visit BPV and covariates.

|  | Intra-dialytic BPV | | | | |  | Visit-to-visit BPV | | | | |
| --- | --- | --- | --- | --- | --- | --- | --- | --- | --- | --- | --- |
| Coefficient | SD | CV | VIM | ARV | Residual |  | SD | CV | VIM | ARV | Residual |
| SD | 1.00 | 0.88 | 0.92 | 0.59 | 0.96 |  | 1.00 | 0.90 | 0.93 | 0.84 | 0.95 |
| CV | 0.88 | 1.00 | 0.99 | 0.46 | 0.85 |  | 0.90 | 1.00 | 0.99 | 0.74 | 0.85 |
| VIM | 0.92 | 0.99 | 1.00 | 0.50 | 0.89 |  | 0.93 | 0.99 | 1.00 | 0.77 | 0.88 |
| ARV | 0.59 | 0.46 | 0.50 | 1.00 | 0.48 |  | 0.84 | 0.74 | 0.77 | 1.00 | 0.87 |
| Residual | 0.96 | 0.85 | 0.89 | 0.48 | 1.00 |  | 0.95 | 0.85 | 0.88 | 0.87 | 1.00 |
| SBP | 0.41 | -0.03 | 0.07 | 0.40 | 0.40 |  | 0.28 | -0.12 | -0.04 | 0.26 | 0.28 |
| Age | 0.15 | 0.21 | 0.20 | 0.13 | 0.14 |  | 0.11 | 0.15 | 0.14 | 0.16 | 0.13 |
| Gender | 0.02 | -0.05 | -0.03 | 0.08 | -0.01 |  | -0.03 | -0.09 | -0.07 | -0.09 | -0.04 |
| Smoking | 0.04 | 0.02 | 0.03 | 0.10 | 0.02 |  | 0.04 | 0.02 | 0.03 | 0.02 | 0.03 |
| Drinking | -0.05 | -0.05 | -0.05 | -0.07 | -0.08 |  | -0.07 | -0.07 | -0.07 | -0.10 | -0.09 |
| DM | 0.27 | 0.18 | 0.20 | 0.20 | 0.24 |  | 0.26 | 0.13 | 0.16 | 0.17 | 0.22 |
| Hyperlipidemia | 0.20 | 0.12 | 0.14 | 0.11 | 0.19 |  | 0.20 | 0.14 | 0.15 | 0.12 | 0.18 |
| CVD history | 0.32 | 0.32 | 0.33 | 0.26 | 0.24 |  | 0.25 | 0.21 | 0.22 | 0.22 | 0.25 |
| Tumor | 0.03 | 0.03 | 0.03 | -0.05 | 0.06 |  | -0.00 | 0.03 | 0.02 | -0.01 | 0.03 |
| Kt/V | -0.12 | -0.05 | -0.07 | -0.02 | -0.10 |  | -0.07 | -0.00 | -0.02 | 0.01 | -0.06 |
| UF | 0.08 | 0.09 | 0.10 | 0.20 | 0.07 |  | 0.08 | 0.01 | 0.02 | 0.05 | 0.04 |
| Dry weight | 0.06 | -0.00 | 0.01 | 0.14 | 0.04 |  | 0.07 | -0.01 | 0.01 | -0.01 | 0.02 |
| Alb | -0.23 | -0.27 | -0.26 | 0.02 | -0.18 |  | -0.11 | -0.16 | -0.15 | -0.16 | -0.15 |
| Hb | -0.21 | -0.21 | -0.21 | 0.07 | -0.23 |  | -0.19 | -0.23 | -0.22 | -0.11 | -0.17 |
| Cr | -0.03 | -0.05 | -0.05 | 0.17 | -0.03 |  | -0.03 | -0.08 | -0.07 | 0.04 | 0.01 |
| K | 0.10 | 0.13 | 0.13 | 0.06 | 0.11 |  | 0.06 | 0.06 | 0.05 | 0.09 | 0.04 |
| Ca | -0.12 | -0.11 | -0.12 | -0.01 | -0.12 |  | -0.02 | -0.02 | -0.03 | 0.05 | 0.02 |
| P | -0.03 | 0.05 | 0.04 | -0.02 | -0.02 |  | -0.07 | -0.02 | -0.03 | -0.05 | -0.04 |
| PTH | -0.01 | -0.02 | -0.01 | 0.15 | -0.01 |  | -0.08 | -0.12 | -0.12 | 0.01 | -0.07 |

**Table S2** Characteristics of high and low CV groups

|  | Intra-dialytic BPV | | | Visit-to-visit BPV | | |
| --- | --- | --- | --- | --- | --- | --- |
| Parameter | Low CV | High CV | P value | Low CV | High CV | P value |
| Sample size | 70 | 50 |  | 65 | 55 |  |
| Male, n (%) | 44 (62.9) | 30 (60.0) | 0.75 | 43 (66.2) | 31 (56.4) | 0.28 |
| Age (years) | 59.59±15.18 | 64.02±12.57 | 0.16 | 59.02±15.31 | 64.29±12.46 | 0.09 |
| SBP (mmHg) | 139.71±16.79 | 138.66±16.55 | 0.88 | 144.28±18.70 | 141.57±14.57 | 0.51 |
| Smoking habit, n (%) | 20 (28.6) | 12 (24.0) | 0.58 | 16 (24.6) | 16 (29.1) | 0.58 |
| Drinking habit, n (%) | 6 (8.6) | 3 (6.0) | 0.60 | 6 (9.2) | 3 (5.5) | 0.44 |
| Comorbidities, n (%)  Diabetes mellitus | 32 (45.7) | 29 (58.0) | 0.19 | 29 (44.6) | 32 (58.2) | 0.14 |
| Hyperlipidemia | 34 (48.6) | 27 (54.0) | 0.56 | 29 (44.6) | 32 (58.2) | 0.14 |
| Hypertension | 57 (81.4) | 46 (92.0) | 0.10 | 52 (80.0) | 51 (92.7) | 0.05 |
| Cardiovascular disease | 30 (42.9) | 29 (58.0) | 0.10 | 27 (41.5) | 32 (58.2) | 0.07 |
| Malignant tumor | 10 (14.3) | 9 (18.0) | 0.59 | 9 (13.8) | 10 (18.2) | 0.52 |
| Dialysis parameters |  |  |  |  |  |  |
| UF (L) | 1.96± 0.65 | 2.17±0.71 | 0.14 | 2.08±0.71 | 2.02±0.65 | 0.53 |
| Dry weight (kg) | 64.39± 10.68 | 65.68±12.61 | 0.44 | 65.30±11.26 | 64.48±11.84 | 0.92 |
| Kt/V | 1.42± 0.26 | 1.39±0.27 | 0.43 | 1.41±0.25 | 1.41±0.28 | 0.89 |
| Laboratory parameters |  |  |  |  |  |  |
| Alb (g/L) | 40.14±2.67 | 38.67±3.45 | 0.02 | 39.79±3.33 | 39.22±2.78 | 0.15 |
| Hb (g/L) | 115.76±11.84 | 109.44±15.41 | 0.02 | 114.96±12.44 | 110.95±14.97 | 0.09 |
| Cr (umol/L) | 858.74± 243.40 | 856.61±278.74 | 0.87 | 884.63±243.76 | 826.20±271.84 | 0.17 |
| BUN (mmol/L) | 26.19±4.94 | 25.80±5.96 | 0.87 | 26.97±4.28 | 24.93±6.28 | 0.06 |
| K (mmol/L) | 4.97±0.65 | 5.23±0.80 | 0.12 | 5.08±0.70 | 5.07±0.76 | 0.96 |
| Ca (mmol/L) | 2.18±0.15 | 2.17±0.18 | 0.84 | 2.17±0.15 | 2.18±0.18 | 0.95 |
| P (mmol/L) | 1.80±0.41 | 1.83±0.51 | 0.29 | 1.84±0.42 | 1.78±0.50 | 0.77 |
| PTH (pg/mL) | 235.03±157.99 | 216.70±145.91 | 0.53 | 239.34±167.36 | 213.28±133.50 | 0.45 |
| TC (mmol/L) | 3.75±0.79 | 4.07±1.01 | 0.11 | 3.79±0.89 | 3.99±0.90 | 0.18 |
| LDL (mmol/L) | 1.85±0.63 | 2.09±0.78 | 0.08 | 1.91±0.70 | 2.00±0.71 | 0.28 |
| BPV metric |  |  |  |  |  |  |
| SD | 13.02±2.42 | 18.78±3.50 | <0.01 | 11.62±2.24 | 17.84±3.88 | <0.01 |
| CV | 0.09±0.01 | 0.14±0.02 | <0.01 | 0.08±0.01 | 0.13±0.02 | <0.01 |
| VIM | 0.05±0.01 | 0.08±0.01 | <0.01 | 0.05±0.01 | 0.08±0.01 | <0.01 |
| ARV | 9.83±2.51 | 11.82±3.18 | <0.01 | 11.94±2.97 | 17.52±4.54 | <0.01 |
| Residual | 9.49±1.79 | 13.80±2.73 | <0.01 | 8.67±1.78 | 13.32±3.27 | <0.01 |
| Outcome |  |  |  |  |  |  |
| CVD event | 16 (0.23) | 26 (0.52) | <0.01 | 22 (0.34) | 20 (0.36) | 0.78 |
| All-cause death | 14 (0.20) | 21 (0.42) | <0.01 | 17 (0.26) | 18 (0.33) | 0.43 |

**Table S3** Univariate Cox regression analysis for confounders

|  | CVD event |  | All-cause mortality |  |
| --- | --- | --- | --- | --- |
| Variable | HR (95% CI) | p-value | HR (95% CI) | p-value |
| SBP | 1.006 (0.988-1.025) | 0.504 | 1.006 (0.985-1.028) | 0.587 |
| Male | 1.815 (0.928-3.550) | 0.081 . | 2.850 (1.240-6.550) | 0.014 * |
| Age | 1.034 (1.008-1.061) | 0.010 ** | 1.042 (1.011-1.074) | 0.008 ** |
| Smoking history | 2.497 (1.334-4.674) | 0.004 ** | 2.014 (1.007-4.026) | 0.048 * |
| Drinking history | 3.247 (1.138-9.264) | 0.028 * | 4.895 (1.851-12.940) | 0.001 ** |
| Diabetes mellitus | 3.348 (1.645-6.815) | 0.001 ** | 1.315 (0.664-2.605) | 0.432 |
| Hyperlipidemia | 2.219 (1.168-4.218) | 0.015 * | 1.269 (0.645-2.498) | 0.490 |
| CVD history | 3.117 (1.618-6.006) | 0.001 ** | 1.971 (0.987-3.940) | 0.055 . |
| Malignant tumor | 1.247 (0.553-2.812) | 0.595 | 2.391 (1.113-5.138) | 0.026 * |
| Ultrafiltration volume | 0.879 (0.573-1.350) | 0.556 | 0.801 (0.493-1.302) | 0.371 |
| Dry weight | 0.998 (0.972-1.024) | 0.862 | 0.996 (0.968-1.025) | 0.801 |
| Kt/V | 0.352 (0.100-1.233) | 0.102 | 0.139 (0.030-0.649) | 0.012 * |
| Alb | 0.961 (0.868-1.065) | 0.446 | 0.904 (0.815-1.003) | 0.057 . |
| Hb | 0.976 (0.955-0.999) | 0.036 * | 0.972 (0.950-0.995) | 0.018 * |
| Cr | 0.998 (0.997-0.999) | 0.002 ** | 0.998 (0.997-0.999) | 0.004 ** |
| K | 1.007 (0.665-1.524) | 0.975 | 0.869 (0.543-1.392) | 0.560 |
| Ca | 0.380 (0.050-2.865) | 0.348 | 0.367 (0.038-3.582) | 0.389 |
| P | 1.026 (0.517-2.038) | 0.941 | 0.780 (0.365-1.666) | 0.521 |
| PTH | 1.000 (0.998-1.002) | 0.840 | 0.998 (0.996-1.001) | 0.194 |
| TC | 1.049 (0.761-1.446) | 0.771 | 0.962 (0.653-1.417) | 0.845 |
| LDL | 1.250 (0.853-1.832) | 0.253 | 1.002 (0.612-1.640) | 0.994 |

Confounders with p-value<0.1 are selected for adjustment. Although ultrafiltration volume and P doesn’t reach significance in the univariate Cox regression analysis, we still consider to include them as representative confounders due to the fact that mineral disorders and regular volume removal are two typical features of dialysis patients. The following confounders are adjusted in Cox proportional hazards model for CVD events: gender, age, smoking history, drinking history, DM, hyperlipidemia, CVD history, Hb, Cr, ultrafiltration volume and P. As for all-cause mortality, gender, age, smoking history, drinking history, tumor, Kt/V, Alb, Hb, Cr, ultrafiltration volume and P are adjusted. ‘.’ indicates p<0.1, ‘*’ indicates p<0.05, ‘**’ indicates p<0.01.

**Table S4** Adjusted Cox regression model for intra-dialytic and visit-to-visit CV

|  | CVD event |  | All-cause mortality |  |
| --- | --- | --- | --- | --- |
| Variable | HR (95% CI) | p-value | HR (95% CI) | p-value |
| **Intra-dialytic CV** | 1.801 (1.224-2.649) | 0.003 ** | 1.282 (0.844-1.950) | 0.244 |
| Male | 3.603 (1.459-8.897) | 0.005 ** | 7.340 (1.902-28.323) | 0.004 ** |
| Age | 1.022 (0.988-1.057) | 0.202 | 1.041 (1.002-1.081) | 0.040 * |
| Smoking history | 2.121 (1.033-4.357) | 0.041 * | 0.762 (0.320-1.816) | 0.539 |
| Drinking history | 2.939 (0.884-9.766) | 0.079 . | 7.771 (2.131-28.337) | 0.002 ** |
| Diabetes mellitus | 1.362 (0.551-3.370) | 0.504 |  |  |
| Hyperlipidemia | 1.137 (0.527-2.454) | 0.743 |  |  |
| CVD history | 1.458 (0.650-3.272) | 0.361 | 1.039 (0.418-2.584) | 0.935 |
| Malignant tumor |  |  | 2.352 (0.936-5.911) | 0.069 . |
| Ultrafiltration volume | 0.492 (0.285-0.850) | 0.011 * | 0.468 (0.240-0.912) | 0.026 * |
| Kt/V |  |  | 0.480 (0.051-4.559) | 0.523 |
| Alb |  |  | 0.949 (0.820-1.010) | 0.488 |
| Hb | 0.978 (0.955-1.002) | 0.076 . | 0.969 (0.945-0.994) | 0.016 * |
| Cr | 0.998 (0.996-1.000) | 0.030 * | 0.998 (0.996-1.000) | 0.052 . |
| P | 2.185 (1.008-4.736) | 0.048 * | 1.240 (0.498-3.090) | 0.644 |
|  |  |  |  |  |
| **Visit-to-visit CV** | 1.476 (0.990-2.200) | 0.056 · | 1.134 (0.750-1.716) | 0.551 |
| Male | 3.149 (1.305-7.600) | 0.011 * | 6.565 (1.799-23.959) | 0.004 ** |
| Age | 1.030 (0.998-1.063) | 0.065 . | 1.043 (1.005-1.082) | 0.025 * |
| Smoking history | 1.923 (0.935-3.955) | 0.076 . | 0.804 (0.343-1.884) | 0.616 |
| Drinking history | 2.392 (0.744-7.691) | 0.143 | 7.232 (2.045-25.574) | 0.002 ** |
| Diabetes mellitus | 1.623 (0.675-3.902) | 0.279 |  |  |
| Hyperlipidemia | 0.982 (0.460-2.100) | 0.963 |  |  |
| CVD history | 1.612 (0.734-3.542) | 0.235 | 1.085 (0.441-2.666) | 0.859 |
| Malignant tumor |  |  | 2.488 (1.010-6.134) | 0.048 * |
| Ultrafiltration volume | 0.535 (0.307-0.932) | 0.027 * | 0.486 (0.250-0.942) | 0.033 * |
| Kt/V |  |  | 0.458 (0.048-4.366) | 0.500 |
| Alb |  |  | 0.949 (0.820-1.097) | 0.478 |
| Hb | 0.979 (0.956-1.003) | 0.085 . | 0.969 (0.944-0.995) | 0.017 * |
| Cr | 0.998 (0.996-1.000) | 0.043 * | 0.998 (0.996-1.000) | 0.062 . |
| P | 2.592 (1.160-5.792) | 0.020 * | 1.402 (0.559-3.520) | 0.472 |

‘.’ indicates p<0.1, ‘*’ indicates p<0.05, ‘**’ indicates p<0.01.
